# Novel therapeutic compound acridine–retrotuftsin action on biological forms of melanoma and neuroblastoma

**DOI:** 10.1007/s00432-018-2776-4

**Published:** 2018-10-26

**Authors:** Miroslawa Cichorek, Anna Ronowska, Monika Gensicka-Kowalewska, Milena Deptula, Iwona Pelikant-Malecka, Krystyna Dzierzbicka

**Affiliations:** 1 Department of Embryology, Medical University of Gdansk, Debinki 1 St, 80-210 Gdansk, PL Poland; 2 Department of Laboratory Medicine, Medical University of Gdansk, Debinki 7 St, 80-211 Gdansk, PL Poland; 3 Department of Organic Chemistry, Gdansk University of Technology, Narutowicza St 11/12, 80-233 Gdansk, PL Poland; 4 Department of Biochemistry, Medical University of Gdansk, Debinki 1 St, 80-210 Gdansk, PL Poland

**Keywords:** Acridine, Retrotuftsin, Melanoma, Neuroblastoma, Apoptosis, Tricarboxylic acid cycle enzymes

## Abstract

**Purpose:**

As a continuation of our search for anticancer agents, we have synthesized a new acridine-retrotuftsin analog HClx9-[Arg(NO_2_)-Pro-Lys-Thr-OCH_3_]-1-nitroacridine (named ART) and have evaluated its activity against melanoma and neuroblastoma lines. Both tumors develop from cells (melanocytes, neurons) of neuroectodermal origin, and both are tumors with high heterogeneity and unsatisfactory susceptibility to chemotherapies. Thus, we analyzed the action of ART on pairs of biological forms of melanoma (amelanotic and melanotic) and neuroblastoma (dopaminergic and cholinergic) with regard to proliferation, mechanism of cell death, and effect on the activity of tricarboxylic acid cycle (TAC) enzymes.

**Methods:**

The cytotoxicity of ART was evaluated by XTT and trypan blue tests. Cell death was estimated by plasma membrane structure changes (phosphatidylserine and calreticulin externalization), caspase activation, presence of ROS (reactive oxygen species), activity of tricarboxylic acid cycle enzymes (pyruvate dehydrogenase complex, aconitase, and isocitrate dehydrogenase), NAD level, and ATP level.

**Results:**

ART influences the biological forms of melanoma and neuroblastoma in different ways. Amelanotic (Ab) melanoma (with the inhibited melanogenesis, higher malignancy) and SHSY5Y neuroblastoma (with cholinergic DC cells) were especially sensitive to ART action. The Ab melanoma cells died through apoptosis, while, with SH-SY5Y-DC neuroblastoma, the number of cells decreased but not as a result of apoptosis. With Ab melanoma and SH-SY5Y-DC cells, a diminished activity of TAC enzymes was noticed, along with ATP/NAD depletion.

**Conclusion:**

Our data show that the biological forms of certain tumors responded in different ways to the action of ART. As a combination of retrotuftsin and acridine, the compound can be an inducer of apoptotic cell death of melanoma, especially the amelanotic form. Although the mechanism of the interrelationships between energy metabolism and cell death is not fully understood, interference of ART with TAC enzymes could encourage the further investigation of its anticancer action.

**Electronic supplementary material:**

The online version of this article (10.1007/s00432-018-2776-4) contains supplementary material, which is available to authorized users.

## Introduction

The acridine family includes a wide range of tricyclic molecules with various biological activities, such as anticancer, antiinflammatory, antimicrobial, antiparasitic, antiviral, and fungicidal activities (Gensicka-Kowalewska et al. 2017; Kukowska 2017). Acridines influence many biological processes, e.g., proliferation, pH homeostasis, and secretion of neurotransmitters. These compounds intercalate between base pairs in the double-stranded DNA structure (Kitchen et al. 1985; Pommier et al. 1987), influence activity of the topoisomerases that control the chromatin structure (Ferguson and Denny 1991) and affect the activity of telomerases and cyclin-dependent kinases (Gunaratnam et al. 2007; Castillo-González et al. 2009; Polewska et al. 2013). Acridine derivatives are inhibitors of acetylcholinesterase and carbonic anhydrase isozymes (Gensicka-Kowalewska et al. 2017; Syrjänen et al. 2014). Many tumors overexpress carbonic anhydrase isoforms which are involved in pH regulation, proliferation, cell migration, and invasion (Mahon et al. 2015; Syrjänen et al. 2014). However, the clinical usefulness of acridine derivatives has thus far been limited due to the risk of high toxicity and tumor resistance (Cen et al. 2018; Wang et al. 2013). Nevertheless, efforts to produce new anticancer acridine derivatives are ongoing (Tang et al. 2012; Othman and Kozurkova 2018). One way of constructing new derivatives is by binding acridine compounds with other biologically active elements, e.g., muramyl dipeptide (MDP), tuftsin, peptide nucleic acids (PNA), or antiinflammatory drugs such as ibuprofen, (*S*)-naproxen, acetylsalicylic acid (Gensicka-Kowalewska et al. 2017; Kukowska 2017), and mycophenolic acid (MPA) (Cholewinski et al. 2016).

Melanoma incidence rates continue to rise worldwide (Rastrelli et al. 2014). Although there has been great progress in immunotherapy based on targets such as CTL4, PD-1, or BRAF, a majority of patients with metastatic or unresectable melanoma still do not survive longer than 5 years (Polkowska et al. 2017; Swe and Kim 2018; Forschner et al. 2017). Thus, there is still a need to develop new chemotherapies against melanoma. This tumor is characterized by great heterogeneity of cells, which seems to be the basic reason for its high chemoresistance (Boeckmann et al. 2011; Searles et al. 2017; Sáez-Ayala et al. 2013). There are some biological forms of this tumor which can be distinguished according to pathomorphological criteria (Rastrelli et al. 2014). One criterion could be the presence of melanin production, which allows distinction between melanotic and amelanotic melanoma cells (Lu et al. 2018). The problem of melanization of melanoma cells, and as a result different susceptibility to the action of cytotoxic drugs, is still an open question in biological research into melanomas (Thomas et al. 2014; Chen et al. 2009; Larsson 1993). Many years ago, it was noticed that *m*-AMSA (aminoacridine), the first synthetic drug to exhibit clinical efficacy as a topoisomerase inhibitor, was effective against melanoma cells. Cells without the presence of melanin were, however, more sensitive to this drug (Stephens and Peacock 1982; Leopold et al. 1987).

**Fig. 1:**
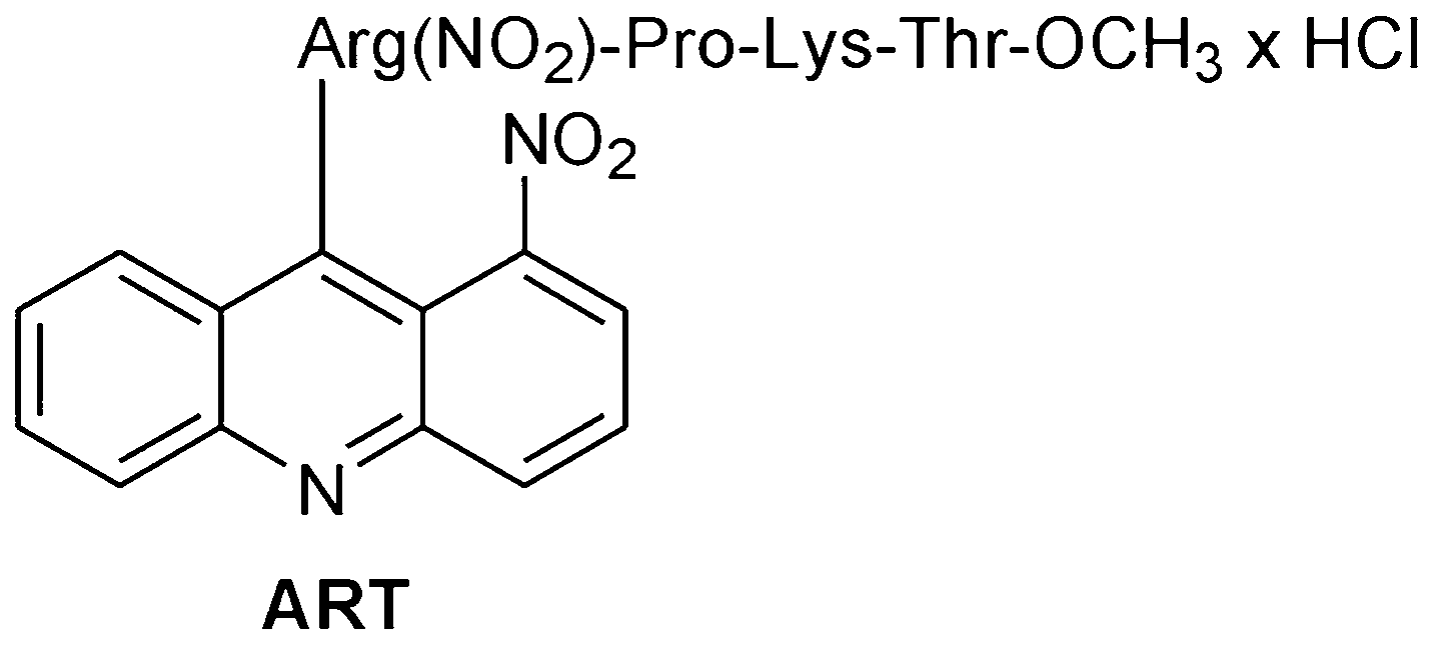
Structure of the synthesized acridine–retrotuftsin analog HClx9-[Arg(NO_2_)-Pro-Lys-Thr-OCH_3_]-1-nitroacridine, named ART

Neuroblastoma is the most common solid childhood tumor outside the brain. It is a tumor type which exhibits different biochemical characteristics of neuronal function and the sensitivity of the cells to different drugs seems to depend on, for example, the synthesis of neurotransmitters (De Ferrari et al. 1998; Svensson 2000; Szutowicz et al. 2006; Chakrabarti et al. 2012; Valter et al. 2018). The SH-SY5Y neuroblastoma line with mainly dopaminergic neurons can be differentiated into cholinergic neurons by in vitro culture, and thus, as a result, it is possible to get two biologically different forms of neuroblastoma (Blusztajn et al. 1987; Szutowicz et al. 2006). Both melanoma and neuroblastoma have some features in common, e.g., neuroectodermal origin of melanocytes and neurons, high heterogeneity, and no sufficiently effective chemotherapies against them. Inspired by the wide range of biological activities of acridine derivatives, and as a continuation of our efforts in search of anticancer agents, we have synthesized new acridine/acridone analogs combined with tuftsin/retrotuftsin derivatives and evaluated them for anticancer activities against melanoma and neuroblastoma lines differing in many biological features (Gensicka-Kowalewska et al. 2018). Tuftsin (tetrapeptide TKPR) is a natural immunomodulator which is present in the blood of humans and other mammals and is capable of stimulating certain white blood cells (monocytes, macrophages, and neutrophils) (Najjar and Nishioka 1970; Fridkin and Najjar 1989; Siebert et al. 2017). Tuftsin derivatives work as anticancer compounds (Noyes et al. 1981; Nishioka et al. 1981; An et al. 2014; Wardowska et al. 2007). Among new analogs synthesized by us, 1-nitroacridine-retrotuftsin (ART; Fig. 1) exhibited the highest anticancer potency. Thus, in this work, we analyzed the mechanism of action of ART on pairs of biological forms of melanoma (amelanotic and melanotic) and neuroblastoma (dopaminergic and cholinergic) regarding proliferation, cell death mechanism, and energetic state.

## Materials and methods

### Transplantable melanomas

The original transplantable melanotic melanoma (Ma) was derived from a spontaneous melanoma of the skin that had appeared in a breed of golden hamster in 1959 (Bomirski et al. 1988). The amelanotic melanoma line (Ab) originated from the Ma form by a spontaneous alteration. The loss of pigment was accompanied by changes in many biological features of the Ab line—faster tumor growth rate, shorter animal survival, and changes in the ultrastructure of cells (Bomirski et al.1988, Sniegocka et al. 2018). Once established, these melanomas possessed a considerable degree of phenotypic stability over decades of passaging (Bomirski et al. 1988). Since their discovery, each melanoma line is maintained in vivo by consecutive, subcutaneous transplantations of tumor material every 21 (Ma) or 11 (Ab) days. The experimental procedures were approved by the Animal Ethics Committee at the Medical University of Gdansk and conformed to the National Health and Medical Research Council’s guide for the care and use of laboratory animals.

### Isolation of melanotic and amelanotic melanoma cells

Melanoma cells were isolated for each experiment from solid tumors by a non-enzymatic method (Zielińska et al. 2008). The suspension consisted of 95–98% viable cells (estimated by trypan blue test).

### Neuroblastoma SH-SY5Y

Neuroblastoma SH-SY5Y is a cloned subline of SK–N–SH human neuroblastoma cells originally established from a bone marrow biopsy of a neuroblastoma patient in the early 1970s. Human SH-SY5Y neuroblastoma cells, between the 10th and 25th passage, were used for experiments. Cells were seeded at a density of 4 × 10^3^ cells/cm^2^ on 25 cm^2^ tissue culture flasks in DMEM medium (Sigma-Aldrich) with 10% FBS (Sigma-Aldrich) and antibiotics (0.05 mg streptomycin and 50 U penicillin per 1 ml; Sigma-Aldrich). Cholinergic differentiation of the DC variant was obtained by combined addition of 1 mmol/L dibutyryl cAMP (cAMP; Sigma-Aldrich) and 0.001 mmol/L all-trans-retinoic acid (RA; Sigma-Aldrich) for 48 h (Bielarczyk et al. 2003). At this time, the medium was replaced by medium without differentiating agents and the test compound ART was added for the next 72 h. SH-SY5Y was purchased from the American Type Culture Collection (ATCC).

### Synthesis of HClx9-[Arg(NO_2_)-Pro-Lys-Thr-OCH_3_]-1-nitroacridine (ART, Fig. 1)

The protected retrotuftsin (Boc-Arg(NO_2_)-Pro-Lys(Fmoc)-Thr-OCH_3_) was synthesized by the conventional chemical procedure using the mixed anhydride method with *N*-methylmorpholine and isobutylchloroformate, isolated by column chromatography and purified by preparative TLC on silica gel (Dzierzbicka et al. 2018; Januchta et al. 2015). The Boc group was removed from the peptide by treatment with trifluoroacetic acid (TFA) to transform it to the trifluoroacetate, which was subsequently used for the synthesis with 1-nitroacridine. The preparation of 1-nitro-9-phenoxyacridine was done by an Ullmann condensation reaction of the potassium salt of *o*-chlorobenzoic acid and *m*-nitroaniline in the presence of copper at 125 °C to give *N*-(3′-nitrophenyl)anthranilic acid, which was refluxed in POCl_3_ to afford the 9-chloro-1-nitroacridine (Ledochowski 1976; Dzierzbicka et al. 2001). This compound was converted by reaction with phenol to 1-nitro-9-phenoxyacridine. A nucleophilic substitution reaction of this product with the TFAxArg(NO_2_)-Pro-Lys(Fmoc)-Thr-OCH_3,_ in phenol at 50 °C under an argon atmosphere gave 9-[Arg(NO_2_)-Pro-Lys(Fmoc)-Thr-OCH_3_]-1-nitroacridine. After completion, ethyl acetate was added and the mixture was extracted with 5% KOH. After evaporation of the solvent, the reaction mixture was purified using preparative thin layer chromatography (TLC) in the solvent CHCl_3_-MeOH (9:1, v/v). The Fmoc-group was then removed using diethylamine (DEA). In the last step, 9-[Arg(NO_2_)-Pro-Lys-Thr-OCH_3_]-1-nitroacridine was dissolved in MeOH and converted to the corresponding hydrochloride using HCl in anhydrous Et_2_O. The product, ART, was characterized by the MS and ^1^H NMR, ^13^C NMR spectra (data not shown, manuscript in press).

### Cell viability assays (XTT, trypan blue)

Cell viability was determined by XTT assay (Roche Diagnostic, USA), which measures the cells’ ability to reduce the tetrazolium salt XTT (2,3-bis-(2-methoxy-4-nitro-5-sulfophenyl)-2H-tetrazolium-5-carboxanilide) to a water-soluble formazan product. Cells were seeded at a density of 5 × 10^3^ neuroblastoma or Ab melanoma and 50 × 10^3^ Ma melanoma cells into 96-well plates with the suitable cultivation media. After 24 h, the media were exchanged and cells were stimulated with appropriate concentrations (1; 10; 20; 40; 50; 100; 150 µM) of ART for 48 and 72 h. The orange-colored formazan product was quantified at 450 nm in a microplate reader (Multiscan FC, Thermoscientific USA). Cell viability was normalized with respect to an untreated control (100%).

In the Trypan Blue test, the cell suspension was mixed with an equal volume of 0.4% isotonic Trypan Blue solution and the total number of cells and fraction of nonviable, dye-accumulating cells was counted after 2 min in a Fuchs–Rosenthal hemocytometer under a light microscope.

### Cytofluorimetric analysis of apoptosis

Flow cytometry analysis was the method used for the estimation of activated caspases, plasma membrane changes (phosphatidylserine and calreticulin externalization), reactive oxygen species (ROS), and cell cycle changes. Cells were seeded in 6-well plates at concentrations of 200 × 10^3^/well for Ab melanoma and 500 × 10^3^ for Ma melanoma, incubated with 100 µM ART for 48 and/or 72 h. Neuroblastoma SH-SY5Y cells were seeded in the culture flasks, differentiating into NC and DC variants, and incubated with 100 µM ART for 72 h. For the comparative apoptosis, analysis cells were also incubated with 9-chloro-1-nitroacridine (A, the basic compound for ART synthesis that has no retrotuftsin), at IC_50_ doses (Ab melanoma 15 µM, both neuroblastoma types 50 µM; unpublished data). For Ma, melanoma determination of an IC_50_ value was not possible, and thus, we used 100 µM as for ART. For the analysis in the flow cytometer, 1 × 10^6^ cells were stained with antibodies conjugated with fluorochrome, or the cells were incubated with a fluorogenic substrate. After incubation, cells were analyzed using a C6 flow cytometer (Becton Dickinson Immunocytometry Systems, USA). After gating out small-sized (e.g. noncellular debris) objects, 10,000 events were collected from each sample. Results were analyzed off-line using the Cyflogic v.1.2.1 software.

### Activated caspases

We used an FLICA test (fluorochrome-labeled inhibitors of caspases) to estimate cells containing activated caspases (Smolewski et al. 2002). The idea of this method is based on the fact that fluorochrome-labeled inhibitors of caspases covalently react with the reactive enzymatic center of activated caspase. We used an FITC (fluorescein)-labeled pan-inhibitor of caspases, FITC-VAD-FMK, which detects most active caspases in the cell. Simultaneous staining with FITC-VAD-FMK and propidium iodide (PI) allows the dynamics of apoptotic death to be followed, by distinguishing the following sequential stages: the early apoptosis C+PI− [have only green fluorescence (active caspases) but excluded PI (plasma membrane is not damaged)]; late apoptosis C+PI+ [bind FITC-VAD-FMK (active caspases) and stained with PI (plasma membrane is damaged)], and in the final stage of apoptosis C−PI+ [cells stained with PI but do not have active caspases].

After 48 and 72 h incubation with ART, the cells were collected and 1 × 10^6^ cells from each experimental point were incubated with 5 µM FITC-VAD-FMK (CaspACE FITC-VAD-FMK, Promega, USA) for 30 min at room temperature in the dark, and then washed and suspended in PBS with 1 µg/ml of PI. Cells were analyzed using a flow cytometer.

### Phosphatidylserine (PS) externalization assay

The early apoptotic change of the plasma membrane structure, PS externalization, was determined by Annexin V-FITC and PI staining (BD Pharmingen, USA) according to the manufacturer’s instructions. The staining allows the determination of populations of cells: viable An−PI−, early apoptotic An+PI− [have only green fluorescence (PS externalization) but excluded PI (plasma membrane is not damaged)]; late apoptotic An+PI+ [have green fluorescence (PS externalization) and stained PI (plasma membrane is damaged)], and final apoptosis/necrosis An−PI+ [absence of green fluorescence (lack of PS externalization) and stained PI (plasma membrane is damaged)]. Fluorescence intensity was measured using a flow cytometer.

### Calreticulin (CRT) externalization assay

The presence of CRT in the plasma membrane characterizes substances that induce the immunogenic type of cell death (Kepp et al. 2011). CRT externalization could work as a signal for immunological cells (Zitvogel et al. 2010). The presence of CRT in the plasma membrane has been determined by an antiCRT antibody conjugated with the fluorochrome phycoerythrin (Abcam, Great Britain). 1 × 10^6^ cells were incubated with antibody diluted 1:100 for 30 min at 4 °C and analyzed in flow cytometer.

### Reactive oxygen species

DCFDA (2′,7′-dichlorofluorescin diacetate) is a fluorogenic dye which measures reactive oxygen species (ROS) activity within a cell. After diffusion into a cell, DCFDA is deacetylated by cellular esterases to a non-fluorescent compound, which is later oxidized by ROS into 2′,7′-dichlorofluorescein (DCF). DCF is a highly fluorescent compound which can be detected by flow cytometry. DCFDA (Sigma-Aldrich) was added to cells according to the manufacturer’s protocol, and after 30 min incubation at 37 °C, the fluorescence intensity was analyzed in a flow cytometer. As a positive control, cells were incubated with 3% H_2_0_2_.(1:30).

### Cell cycle analysis

Cell cycle distribution was determined by the flow cytometry method based on the DNA content in cells’ nuclei, as described earlier (Cichorek 2011). Ethanol-fixed cells (1 × 10^6^) were resuspended in 1 ml of a staining solution containing 40 µg/ml propidium iodide (Sigma Chemicals, USA) and 100 µg/ml RNaze A (Sigma Chemicals, USA), and incubated for 30 min at 37 °C. Results were analyzed by flow cytometry.

### Immunoblotting

Total cell lysates were obtained by incubation of 3 × 10^6^ cells in the lysis buffer RIPA (Sigma-Aldrich, USA), for 1 h on ice, followed by spinning for 15 min at 14,000 rpm. The supernatants were collected and stored at − 70 °C until further processing. The total protein amount was quantified by Bradford assay (Sigma-Aldrich, USA). 60 µg of the lysates were subjected to electrophoresis in 15% SDS gel under reducing conditions and then transferred to a nitrocellulose membrane (Bio-Rad, USA). After 2 h blocking in 4% non-fat milk, membranes were probed overnight with the primary antibody mouse monoclonal anticaspase 9 clone 5B4 (Enzo Life Science, USA; 1:1000). Antiβ-actin mouse monoclonal antibody (Sigma Chemicals, USA; 1:15 000) was used as an equal protein loading control. After washing, the membranes were incubated for 2 h with horseradish peroxidase-conjugated secondary antibody (Sigma-Aldrich, USA, USA; 1:10,000). A chemiluminescent signal was developed using the Super Signal West Pico system (Thermo Scientific, USA) and documented by the Chemi Doc XRS + imaging system (Bio-Rad). Band intensity was semiquantified using the ImageLab 5.2.1 software (National Institutes of Health, USA).

### Activity of enzymes connected with energetic state of cells

PDH (pyruvate dehydrogenase complex), aconitase, and NADP-isocitrate dehydrogenase (IDH) activities were estimated in cell lysates after 48 and 72 h culture with ART. Cells were harvested into ice cold Puck’s solution (140 mmol/l NaCl, 5 mmol/l KCl, 5 mmol/l glucose, 1.7 mmol/l Na-phosphate buffer, and pH 7.4), centrifuged at 200 g for 7 min, and suspended in 0.320 mmol/l sucrose buffered with HEPES (pH 7.4) and 0.1 mM EDTA-Na. Protein levels were measured using the Bradford method, with human immunoglobulin as standard. The protein concentration in suspension was about 2 mg/ml in Ab and 5 mg/ml in SH-SY5Y. The cells were counted using Trypan Blue assay and the Tricarboxylic acid cycle (TCA) enzymatic activities were measured. For PDH activity, the cells were frozen at − 20 °C for up to 5 days.

Before the assay, samples were diluted to the desired protein concentration in 0.2% Triton X-100. PDH activity was assayed by the citrate synthase coupled method followed by citrate quantisation using citrate lyase. Aconitase and IDH activity were assayed by a direct measurement of NAD reduction. The influence of ART on enzyme activities is presented as the half maximal inhibitory concentration (IC_50_).

### Energetic and oxidoreduction potential of cells

Energetic states were assayed by determining the content of ATP and NAD in cell extracts by high-performance liquid chromatography (HPLC) with mass detection (Slominska et al. 2006).

### Statistical analysis

The statistical analysis was performed using the data analysis software system STATISTICA version 12, StatSoft Inc. (2016). Data are expressed as an arithmetical mean ± standard deviation (SD) or standard error of the mean (SEM). To compare the differences between examined groups, results were analyzed by nonparametric Mann–Whitney *U* test, in which *p* < 0.05 was considered statistically significant.

## Results

### Cell viability (XTT, trypan blue)

**Fig. 2:**
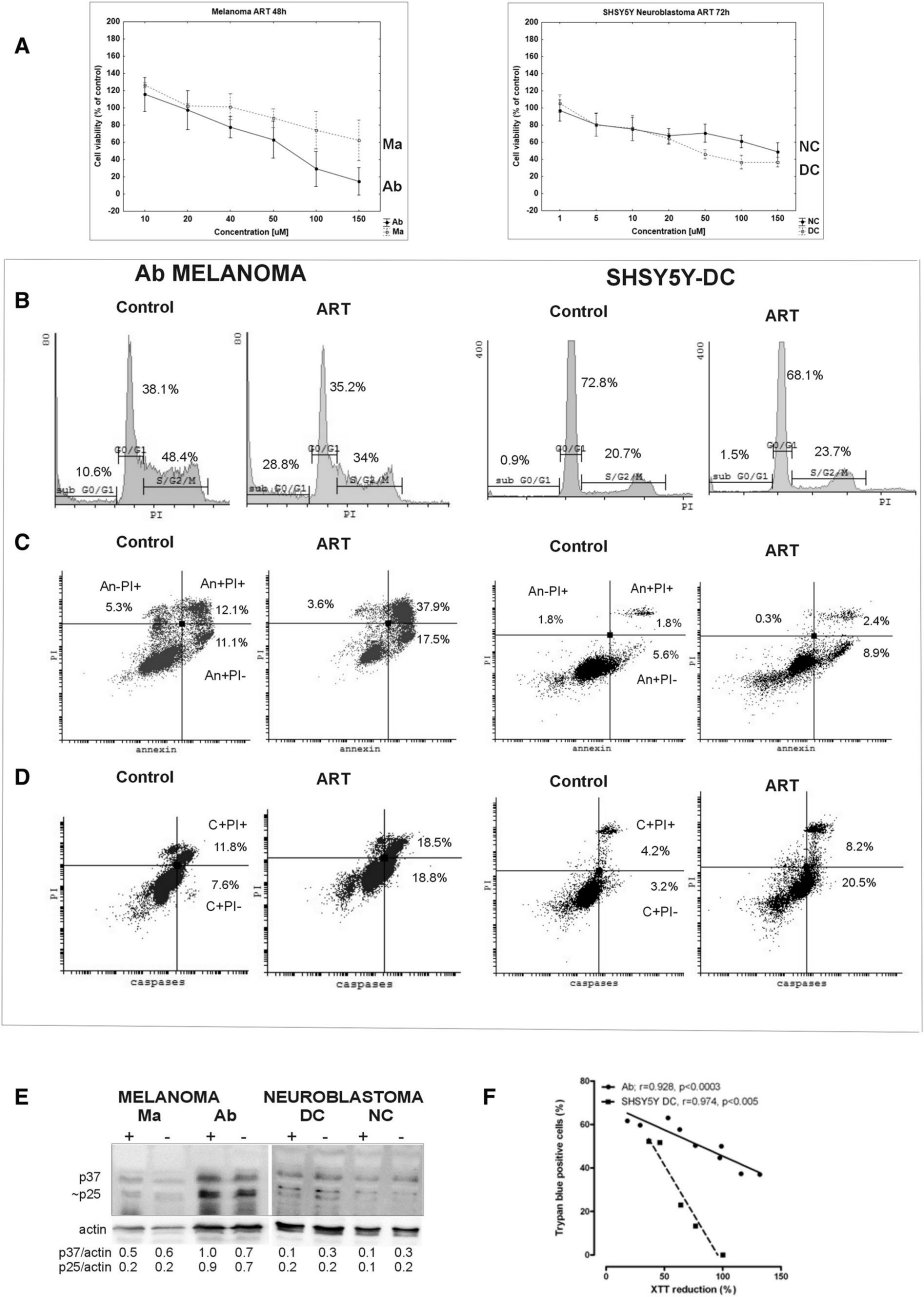
Elements of the potential mechanism of ART cytotoxicity and ways of cell death (A–D for Ab cells after 48 h with ART; A–D for SH-SY5Y-DC after 72 h with ART). **a** Cytotoxicity XTT assay results of at least three experiments expressed as mean ± SD; **b** Example of the cell cycle analysis, showing percentage of cells in cell cycle phases: G0/G1, S/G2/M, and cells with decreased DNA content, e.g., apoptotic bodies, located in subG0; **c** Example of the plasma membrane apoptotic change in structure–PS externalization results analysis [percentage of cells with early apoptotic (An+PI−), late apoptotic (An+PI+) and necrotic (An−PI+)] plasma membrane changes; **d** example of results concerning analysis of caspase activation (percentage of cells with the early apoptotic (C+PI−) and late apoptotic (C+PI+) changes with regard to caspase activation); **e** activation of procaspase 9, an intrinsic way of apoptosis induction as evidenced by the presence of p37 and p25 proteins, products of procaspase proteolytic activation; **f** correlation between trypan blue stained cells (TB+) and XTT reduction in Ab melanoma and SH-SY5-DC neuroblastoma cells

**Fig. 3:**
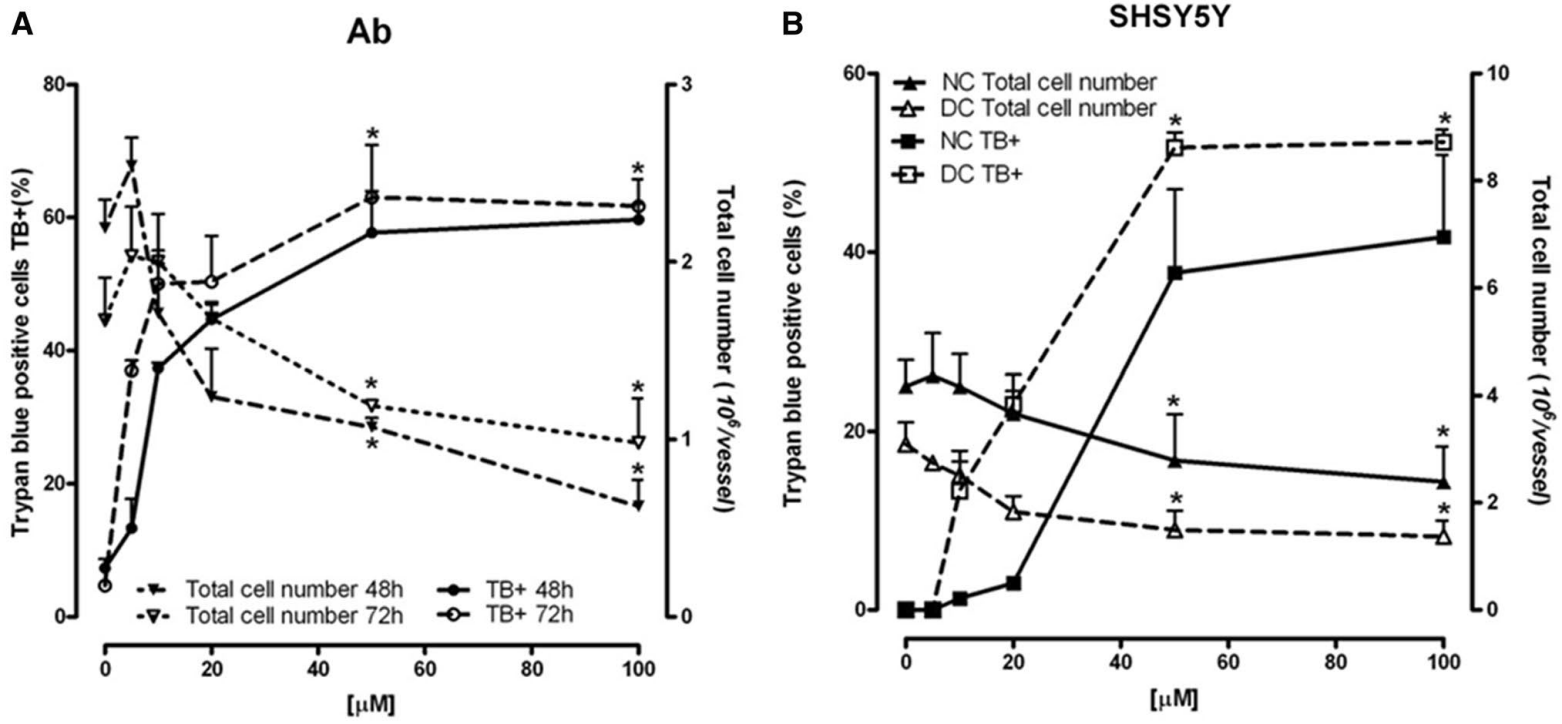
Concentration-dependent effect of ART on TB+ and total cell number in Ab cells (**a**) and nondifferentiated (NC) and differentiated (DC) SH-SY5Y cells (**b**). Data are given as mean ± SEM from three to four experiments. Significantly different from respective control *p < 0.05

ART was cytotoxic against melanoma and neuroblastoma cells in a dose-dependent manner, but different biological forms of both tumors responded in different ways when the dose increased over 20 µM (Fig. 2a). Among melanomas, the more sensitive type was amelanotic Ab melanoma, which after just 48 h incubation with 50 µM of ART had over 30% of cells with inhibited mitochondrial activity (Fig. 2a) and 60% stained with trypan blue dye (TB+; Fig. 3a). Under the same conditions, only 12% of melanotic Ma melanoma showed mitochondrial changes (Fig. 2a), and TB+ cells were absent (data not shown). The same ART concentration but only after 72 h induced mitochondrial activity inhibition in 50% of DC and over 20% of NC neuroblastoma cells (Fig. 2a), which over 50% of DC neuroblastoma cells and 40% of NC neuroblastoma cells were TB+ (Fig. 3b). The observed cytotoxic ART action on Ab melanoma, and on neuroblastoma cells (especially the DC line), was accompanied by a cell number decrease (Fig. 3).

### Cell cycle changes

There were 24% and 38% cells in S/G2/M phases among Ma and Ab melanoma lines, respectively (Table 1). Cells in the G0/G1 phase comprised 56% and 44% of Ma and Ab cells, respectively. After incubation with ART, the percentage of Ab melanoma cells had decreased significantly, after just 48 h, to 26% in S/G2/M phases and to 30% in the G0/G1 phase. These changes were accompanied by a statistically significant increased number of cells in the sub-G0 area, which comprised over 40% of all Ab cells (Table 1; Fig. 2b). Thus, under the influence of ART, Ab melanoma cells were damaged into fragments with decreased DNA content (sub G0 area), and dying cells came from all cell cycle phases. Under the same conditions, but with prolonged incubation with ART to 72 h, Ma melanoma cells showed a slight decrease to 15% and 49% cells in S/G2/M phases and the G0/G1 phase, respectively. This was accompanied by an increase of cells in the sub-G0 area also (Table 1).

**Table 1:** Cell cycle analysis and cells with decreased DNA content (sub G0) after incubation without (control) and with ART (100 µM) after 48 h and 72 h among melanoma (melanotic Ma, amelanotic Ab) and neuroblastoma SH-SY5Y (NC, DC) lines

Cell line	Percentage of cells in particular cell cycle phases				Sub G0	
	G0/G1		S/G2/M			
Incubation time in hours	48	72	48	72	48	72
Ma melanoma						
Control	55.9 ± 11.7	53.3 ± 8.5	24.3 ± 4.2	19.4 ± 3.2	16.0 ± 7.7	23.8 ± 11.7
ART	50.2 ± 22.7	48.5 ± 20.0	18.2 ± 3.2*	14.7 ± 2.0*	28.6 ± 25.6	33.1 ± 21.3
Ab melanoma						
Control	43.6 ± 6.0	42.8 ± 8.6	38.0 ± 8.3	28.0 ± 8.1	16.5 ± 7.1	25.6 ± 9.5
ART	30.2 ± 5.0*	24.3 ± 13.1*	25.7 ± 6.8*	17.6 ± 11.1	41.3 ± 8.8*	54.4 ± 20.4*
SHSY5Y NC						
Control	ne	82.7 ± 9.5	ne	14.1 ± 7.8	ne	0.6 ± 0.3
ART	ne	74.9 ± 12.4	ne	19.7 ± 8.6	ne	1.6 ± 0.5
SHSY5Y DC						
Control	ne	77.6 ± 9.3	ne	18.3 ± 7.6	ne	0.9 ± 0.6
ART	ne	71.2 ± 12.1	ne	22.6 ± 7.0	ne	1.4 ± 0.5

Values are given as the mean ± SD. Statistical analysis by *U* Mann–Whitney test

*Statistically significant change (*p* < 0.05) in comparison to control values. For Ma 3–5, Ab 7–13, DC 5–8, NC 3–8 experiments were done. ne not estimate

There were 14% and 18% cells in S/G2/M phases among NC and DC neuroblastoma cells, respectively. Cells in the G0/G1 phases comprised 83% and 78%, respectively, for the NC and DC lines. After incubation with ART, these values did not change significantly as the sub-G0 area included only about 1% of all cells (Table 1; Fig. 2b). Thus, ART did not influence the neuroblastoma cell cycle and did not induce cell fragmentation into apoptotic bodies.

### Plasma membrane structure changes in dying cells

ART induced changes in the plasma membrane structure such as the externalization of phosphatidylserine (An+ cells) but without calreticulin presence in it. After 48 h, over half of Ab melanoma cells were annexin-positive, of which 24% were An+PI− (early apoptotic) and 30% were An+PI+ (late apoptotic) cells with a discontinuous plasma membrane that allows nucleus staining with PI (PI+). With prolonged incubation time to 72 h, the content of An+PI− cells increased to 35%, while An+PI+ did not change significantly in comparison to cells not treated with ART (Table 2; Fig. 2c). Under the same culture conditions, after 72 h, the number of An+PI− and An+PI+ Ma melanoma cells slightly increased to 20% and 13%, respectively (Table 2). ART does not increase the number of necrotic cells (An−PI+) among both melanoma lines and these cells were at a level of 2–5% (Table 2). ART does not significantly influence phosphatidylserine externalization in both neuroblastoma lines (Table 2; Fig. 2c). However, there is a tendency toward a slight increase of An+PI− cells to 8% after ART action (Table 2; Fig. 2c). Incubation with ART does not induce calreticulin translocation to the plasma membrane of any of the examined tumor line cells (Table 2).

**Table 2:** ART action on melanoma (melanotic Ma and amelanotic Ab) and neuroblastoma (dopaminergic NC and cholinergic DC) cells referring regulated cell death features as caspases activation, phosphatidylserine, and calreticulin externalization, ROS production

	Melanoma								Neuroblastoma SHSY5Y			
Percentage of cells with	Melanotic Ma				Amelanotic Ab				NC		DC	
	48 h		72 h		48 h		72 h		72 h		72 h	
	Control	+ART	Control	+ART	Control	+ART	Control	+ART	Control	+ART	Control	+ART
1. Activated caspases												
All C+	7.2. ± 6.6	9.8 ± 2.4	5.5 ± 4.2	11.4 ± 5.2	9 ± 4.2	31.5 ± 7.1*	16.3 ± 6.2	30.3 ± 9.9*	5.5 ± 2.5	16.0 ± 8.2*	7.0 ± 2.7	27.2 ± 2.7*
Early apoptotic C+PI−	1.8 ± 2.6	2.8 ± 0.7	4.0 ± 4.8	3.3 ± 2.5	2.2 ± 1.9	11.2 ± 5.8*	4.1 ± 3.4	15.9 ± 10.2*	1.7 ± 0.7	9.3 ± 6.1	2.3 ± 1.1	16.7 ± 9.9*
Late apoptotic C+PI+	5.9 ± 5.1	7.1 ± 1.7	4.0 ± 2.8	8.1 ± 3.0	6.8 ± 2.8	20.4 ± 3.2*	12.2 ± 4.1	14.4 ± 4.7	3.9 ± 2.0	6.7 ± 3.9	4.4 ± 1.3	9.4 ± 3.7*
2. Phosphatidylserine externalization												
Early apoptotic An+/PI−	16.8 ± 8.1	20.9 ± 9.4	14.8 ± 6.2	**20.6 ± 6.3**	10.6 ± 5.7	24.4 ± 5.6*	11.8 ± 7.2	35.3 ± 13.9*	3.8 ± 2.5	8.5 ± 3.1	4.4 ± 2.2	8.8 ± 4.4
Late apoptotic An+/PI+	7.9 ± 3.7	11.8 ± 3.1	7.0 ± 3.9	13.3 ± 3.6	13.9 ± 6.6	29.8 ± 6.8*	21.8 ± 7.5	22.9 ± 5.6	3.5 ± 1.7	4.8 ± 3.1	2.4 ± 0.7	5.7 ± 4.0
Final apoptotic/Necrotic An−/PI+	4.0 ± 4.3	4.6 ± 4.2	2.3 ± 1.7	3.8 ± 0.8	5.3 ± 2.6	3.2 ± 1.5	3.7 ± 2.3	4.1 ± 2.9	0.4 ± 0.3	1.6 ± 1.8	0.4 ± 0.2	4.6 ± 6.7
3. Calreticulin (CRT) externalization	1.3 ± 0.7	2.0 ± 0.2	2.7 ± 3.5	6.9 ± 3.2	5.2 ± 4.9	1.1 ± 0.4	4.4 ± 2.5	2.4 ± 1.8	1.1 ± 0.7	0.3 ± 0.3	1.0 ± 0.5	0.4 ± 0.3
4. Reactive oxygen substrates (ROS)	42.9 ± 8.2	39.9 ± 5.0	35.9 ± 12.1	42.7 ± 6.9	47.9 ± 23.6	30.3 ± 9.3	42.8 ± 20.4	22.2 ± 9.1	80.7 ± 15.3	49.2 ± 9.2*	78.2 ± 13.5	51.0 ± 11.2*

Values are means ± SD from 3 to 13 experiments (Ma 3–5, Ab 7–13, DC 5–8,NC 3–8). Statistical analysis by *U* Mann–Whitney test; * Statistically significant change (*p* < 0.05) in comparison to control values

### Caspase activation

Among melanoma lines, ART significantly increased the content of cells with activated caspases only in Ab melanoma cells. After 48 h 32% of Ab melanoma cells have activated caspases (C+), of which 11% were C+PI− (early apoptotic) and twofold more were C+PI+ (late apoptotic). After 72 h, the content of C+PI− cells reaches 16%, while C+PI+ does not change significantly in comparison to cells not treated with ART (Table 2; Fig. 2d). Under the same culture conditions, after 72 h, 3% of Ma melanoma cells were C+PI− and 8% of C+PI+ cells, similar to control cells incubated without ART (Table 2).

Among neuroblastoma cells, ART significantly increased the content of caspase-positive cells to 27% and 16% for DC and NC, respectively. The early apoptotic C+PI− cells dominated among these cells and comprised 3/5th of caspase-positive cells (Table 2; Fig. 2d).

Western blot results confirmed that among the activated caspases was caspase 9 (as indicated by the presence of the p37 and 25 proteins after ART action), an enzyme which plays a critical role in induction of apoptosis (Fig. 2e).

### ROS activation

Both melanoma lines show about 40% of cells with ROS activity. Under influence of ART, these values did not change in Ma melanoma cells, but, in Ab melanoma, it decreased to 22% after 72 h (Table 2). There were 80% of ROS-positive cells among neuroblastoma cells, much more than in the melanoma lines. Incubation with ART decreased this percentage to 50% in both neuroblastoma lines (Table 2).

To sum up, in tests on the activity of ART on biological forms of the examined melanomas and SH-SY5Y neuroblastoma cells, amelanotic Ab melanoma (with inhibited melanogenesis) and SH-SY5Y-DC (with dominating cholinergic phenotype of cells) were especially sensitive. Cells of these sensitive lines react in different ways to ART action. It was observed that Ab melanoma cells died through apoptosis (caspase activation and plasma membrane changes), while, with SH-SY5Y-DC, neuroblastoma cell death was marginal (with a significant caspase activation). Decreasing number of these latter cells thus seemed to be the result of a cytostatic, and not cytotoxic, action of ART. ART-induced decreased ability to reduce the tetrazolium salt XTT by mitochondria correlates with trypan blue-positive (TB+) cells in tested tumor lines (Fig. 2f).

ART (9-RT-1-nitroacridine) was more effective in inducing apoptotic cell death than the basic compound A (9-chloro-1-nitroacridine) (Supplementary Tables 1 and 2).

Thus, as the next step of our experiment, we followed the some elements of the energetic metabolism of examined cells after ART action.

### Activity of enzymes connected with the energetic state of cells

#### Pyruvate dehydrogenase complex (PDHC)

**Fig. 4:**
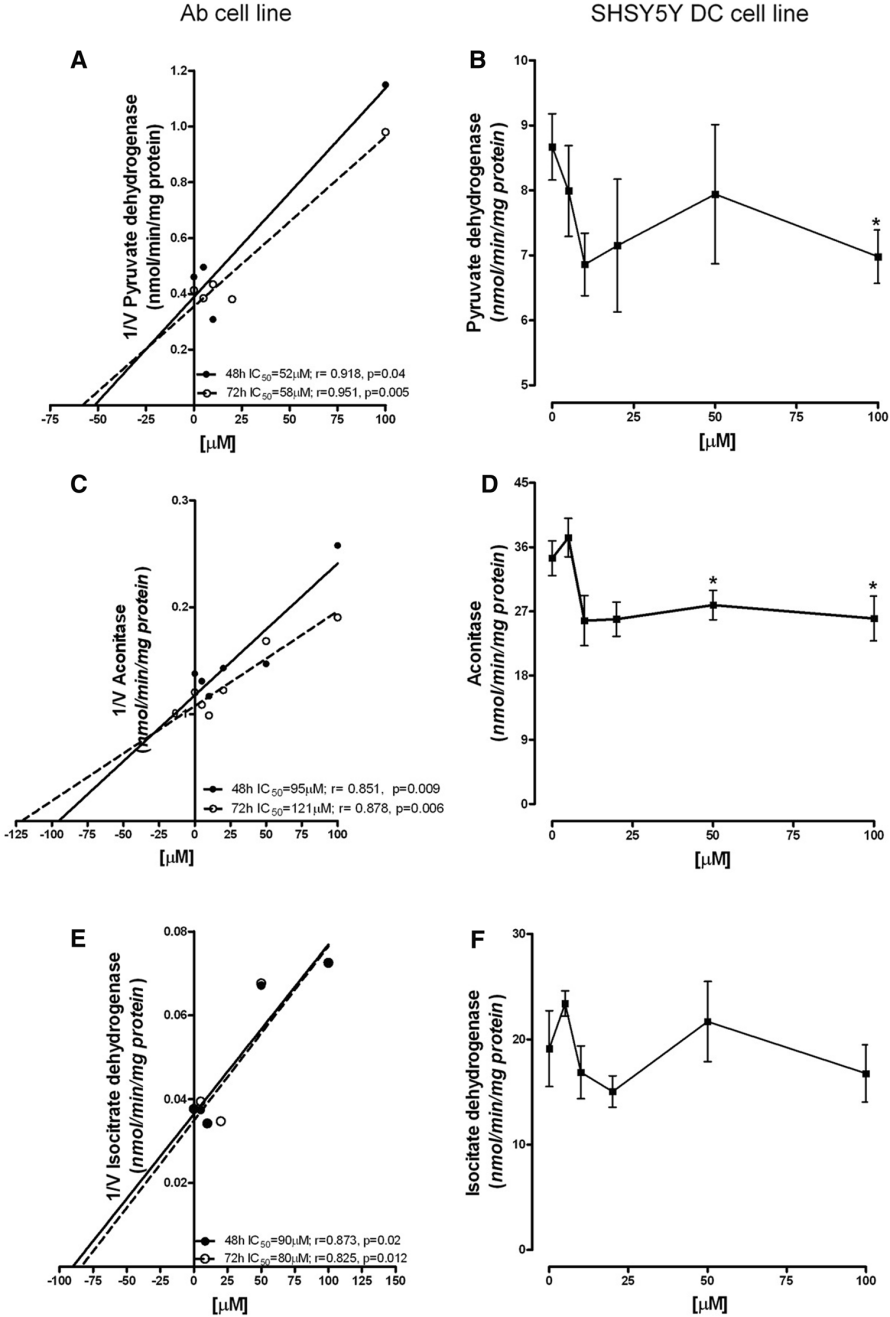
Concentration-dependent effect of ART on TCA cycle enzyme activities: Dixon plot of PDHC in Ab cells (**a**); PDHC activity in SH-SY5Y-DC (**b**); Dixon plot of aconitase in Ab cells (**c**); aconitase activity in SH-SY5Y-DC (**d**); Dixon plot of IDH in Ab cells (**e**); IDH activity in SH-SY5Y-DC cells (**f**). Data are given as mean ± SEM from three to four experiments. Significantly different from respective control *p < 0.05

The activity of PDHC in control Ab cells was 2.43 ± 0.15 nmol/min/mg protein. It was inhibited by ART in a concentration-dependent manner, with the IC_50_ at 48 h being 52 µM; longer incubation did not significantly change this effect, and the IC_50_ at 72 h was 58 µM (Fig. 4a). SH-SY5Y-DC exhibited sensitivity to ART only after 72 h of incubation (Fig. 4b). The activity of PDHC in these cells was inhibited by 20% after treatment with 10 µM of ART. A further increase in the ART concentration did not increase the inhibitory effect (Fig. 4b). The activity of the enzyme in the control was 8.67 ± 0.51 nmol/min/mg protein (Fig. 4b).

#### Aconitase

The activity of aconitase in control Ab cells after 48 h was 7.25 ± 0.37 nmol/min/mg protein. ART strongly inhibited the activity of aconitase, after both 48 h and 72 h, in Ab cell cultures, with an IC_50_ of 95 µM after 48 h. Further incubation to 72 h did not significantly influence the inhibitory effect (Fig. 4c). SH-SY5Y-DC showed aconitase activity of 34.45 ± 2.43 nmol/min/mg protein under control conditions (Fig. 4d). ART, added to the cell culture for 72 h at a concentration 10 µM, decreased this enzyme’s activity by 30%. Further increase of ART concentration did not increase the inhibitory effect (Fig. 4d).

#### Isocitrate dehydrogenase (IDH)

The activity of IDH in Ab cells after 48 h, under control conditions, was 26.40 ± 2.46 nmol/min/mg protein. ART, added to the cell culture for 48 h or 72 h, inhibited the activity of IDH, with an IC_50_ of about 90 µM after both 48 h and 72 h (Fig. 4e). The activity of IDH in SH-SY5Y DC cultures, under control conditions, was 19.12 ± 3.60 nmol/min/mg protein. ART added for 72 h did not inhibit IDH activity in SH-SY5Y-DC cells (Fig. 4f).

#### NAD and ATP

**Fig. 5:**
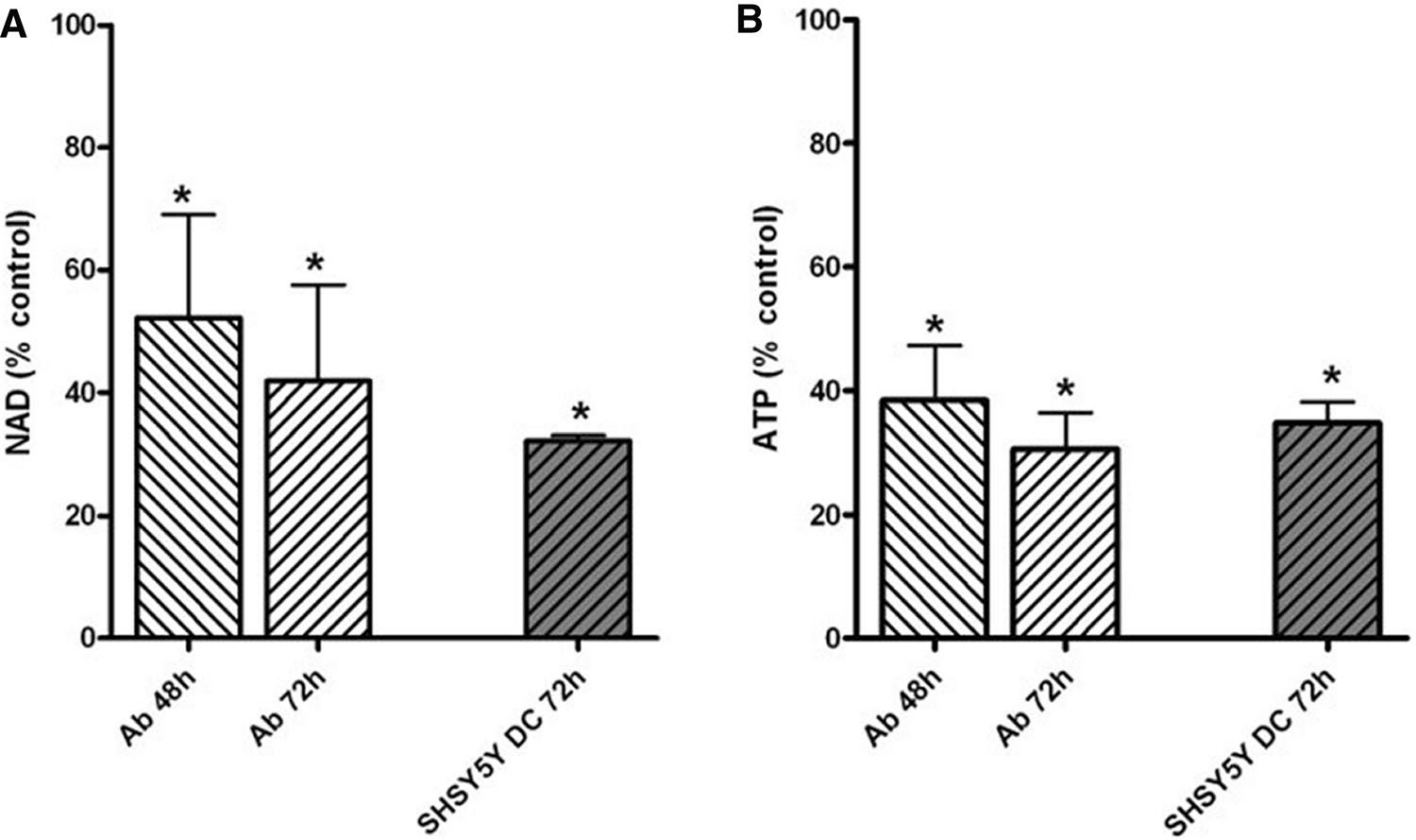
Effect of ART on: NAD (**a**) and ATP (**b**) levels in Ab cells and SH-SY5Y-DC cells. Data are given as mean ± SEM from 4 experiments. Significantly different from respective control; *p < 0.05

The NAD level was decreased by 48% after 48 h and 60% after 72 h incubation with 100 µM ART in Ab cells (Fig. 5a), whereas the ATP level was respectively depleted by 60% and 70% after incubation with ART for these times (Fig. 5b). There was about a 65% decrease in NAD (Fig. 5a) and a 55% decrease of ATP level in SH-SY5Y-DC cells after 72 h of incubation with 100 µM ART (Fig. 5b).

## Discussion

Our results indicate that ART doses over 20 µM influence, in different ways, different biological forms of melanoma and neuroblastoma. Amelanotic melanoma, which showed inhibited melanogenesis, higher growth rate and higher malignancy, and neuroblastoma with dominating cholinergic cells, were especially sensitive to ART action.

The observed increase of damaged cells (located in the sub-G0 area), and decreasing numbers of cells in the G0/G1 and S/G2/M phases, indicated that ART induced Ab melanoma cell death independently of the cell cycle phase. Amelanotic melanoma has greater proliferating potential (38% cells in S/G2/M phases) in comparison to neuroblastoma (only 18% cells in S/G2/M phases), but we did not observe the cell cycle arrest of any phases among cells of both lines. However, acridines, as agents interacting with chromatin structures, have caused cell cycle arrest followed by cell death in many tumor lines (Su et al. 1992; Antonini 2002; Kukowska-Kaszuba et al. 2007; Polewska et al. 2013; Skladanowski 2002).

Among SH-SY5Y-DC, the content of dead cells was marginal, only about 1% located in the sub-G0 area, although ART caused inhibition of mitochondrial activity among similar numbers of cells as in the Ab line, as measured by XTT assay, and the number of cells decreased with time.

According to the recommendations of the Committee on Cell Death (Galluzzii et al. 2018) in our work, we have followed the elements of cell death machinery that could be regulated by ART action, for example, plasma membrane structure changes that could influence the immunological system (immunogenic cell death; eat me signals such as calreticulin expression), caspase activation, energetic state (ATP and NAD levels), and activity of metabolic enzymes, particularly tricarboxylic acid cycle enzymes.

Calreticulin is one of the molecules named alarmins or DAMPs (damage associated molecular patterns). It is translocated from the endoplasmatic reticulum to the plasma membrane of tumor cells under the influence of a currently limited number of chemotherapeutics, as an early apoptotic event (Obeid et al. 2007) also in melanoma cells (Dudek-Peric et al. 2015).

Unfortunately we did not observe calreticulin externalization in the examined cells after ART action, although such an effect has been noticed with melphalan as antimelanoma drug (Dudek-Peric et al. 2015).

In the Ab melanoma line, ART-induced mitochondria inhibition was followed by cell apoptosis, while, in SH-SY5Y-DC, it was not. After 48 h, 70% of Ab cells had inhibited mitochondrial activity and cells with late apoptotic features dominated (as evidenced by caspase activation and phosphatidylserine externalization accompanied by the loss of plasma membrane integrity that allows PI enter into a cell). SH-SY5Y-DC cells needed an additional day to get the caspase-positive cells to a level similar to that observed in amelanotic Ab melanoma cells. While among Ab melanoma annexin-positive cells reached about 50%, among neuroblastoma cells, even after the prolonged time of incubation with ART, the plasma membrane changes were occurring only in a small portion, 15%, of cells. Thus, the aforementioned results for the apoptosis dynamic nicely illustrate that, in Ab melanoma cells, this type of cell death occurred faster than in neuroblastoma cells.

To sum up, the decreasing number of Ab melanoma cells observed in the presence of ART was the result of cytotoxic activity through regulated cell death—apoptosis—as evidenced by an increased number of cells with the activated caspases and extracellular phosphatidylserine localization. In contrast, among SH-SY5Y-DC neuroblastoma cultures, the decreasing number of cells reflected the inhibition of proliferation of these cells, not cell death.

Among both examined lines, we observed changes in the activities of enzymes involved in cell energetic metabolism. Cell death in all its forms is associated with a bioenergetic and redox crisis that may constitute its actual cause (Galluzzi et al. 2018; Valter et al. 2018). On the other hand, one well-known hallmark of any cancer is reprogramming of energy metabolism. Non-tumor cells in physiological conditions, in the presence of oxygen, convert glucose to pyruvate in a glycolytic pathway, and this is further metabolized in the TCA cycle in mitochondria. However, under hypoxic conditions, glycolysis is overactivated which leads to limitations in pyruvate utilization in mitochondria. In cancer cells, there is a shift in energy metabolism from aerobic to anaerobic and energy metabolism largely depends on glycolysis, a state named “aerobic glycolysis” which was characterized first by Warburg and is named the Warburg effect (Warburg et al. 1958). As a consequence, lactic acid fermentation is activated, but, through this pathway, less ATP is produced to supply energy needs. This is compensated for by a higher glucose influx to make up for inefficient ATP synthesis in cancer cells (Maus and Peters 2016). However, the newest research postulate that cancer cells are able to use aerobic glycolysis to support the process of biosynthesis (Vander Heiden et al. 2009).

The mechanism of acridine toxicity is still not well understood (Denny 2004; Kukowska 2017; Cholewiński et al. 2016). One possible way that acridines act could be the inhibition of the activity of key enzymes of the tricarboxylic acid cycle (TCA). ART led to inhibition of PDHC, especially in the Ab cell line and also in SH-SY5Y-DC cells. The latter are characterized by stronger susceptibility to cytotoxic insults. This observation may be explained by the fact that differentiation of the SH-SY5Y cells is conducted towards the cholinergic phenotype. Highly differentiated cholinergic neurons are more sensitive to any cytotoxic factors (Szutowicz et al. 2006, 2013). This phenomenon is explained by the fact that SH-SY5Y-DC use acetyl-CoA, a product of PDHC reaction, in the TCA and in acetylcholine synthesis. As a consequence, the other enzymes of the TCA might also be limited more strongly than in SH-SY5Y-NC line. The antiacetylcholine action of acridine derivatives, e.g., tacrine, has been observed by others (Svensson 2000; De Ferrari et al. 1998).

**Fig. 6:**
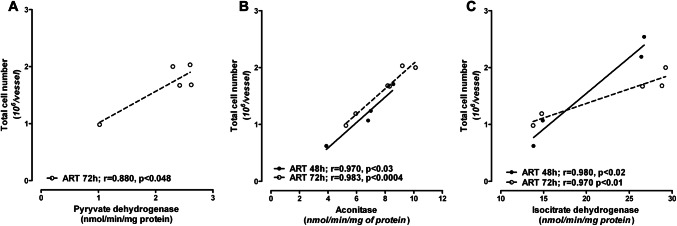
Correlation between chosen TCA enzyme activities and the total cell number in the Ab cell line

Inhibition of the activities of PDHC, aconitase and isocitrate dehydrogenase by ART in Ab cells could be responsible for the total cell fall as a result of apoptotic death. The strong correlation between the activities of these enzymes and total cell number supports this statement (Fig. 6).

Several hallmark cancer mutations have been observed to occur in metabolic enzyme genes (King et al. 2006) to which, more recently, isocitrate dehydrogenase has been added (Sjöblom et al. 2006; Parsons et al. 2008; Kang et al. 2009). Multiple preclinical models have provided evidence for the oncogenic potential of isocitrate dehydrogenases, which alter cancer cell differentiation and metabolism (Kroemer and Pouyssegur 2008; Vander Heiden et al. 2009; Yen et al. 2010; Dang et al. 2016). Mutations within these genes have been found in many cancers (Dang et al. 2016; Sjöblom et al. 2006; Parsons et al. 2008; Abbas et al. 2010) among which are melanoma (Shibata et al. 2011; Lopez et al. 2010). The mutated enzyme catalyzes NADP+/NAD+-dependent conversion of isocitrate to 2-hydroxyglutarate (2HG), while the wild type converts isocitrate to α-ketoglutarate. As a consequence this metabolite accumulates in the cell and is further able to affect the demethylating process in the genome (Maus and Peters 2017). Thus 2HG is also known to be an “oncometabolite”. It therefore looks as if the mutation is a cause of a tumour, not a side effect, and this enzyme could thus be a target for anticancer compounds. Although IDH activity in the other tested lines did not exhibit susceptibility to ART, there is a trend toward decreasing this enzyme’s activity in highly differentiated cells (Fig. 4f). Differing activities of IDH in Bomirski hamster melanomas has been observed previously (Scislowski and Slominski 1983).

It is noticeable that, in all cell types, there was a total ATP decrease. Although Ab cells were again revealed to be more susceptible (Fig. 5), the reason for the fall is probably caused by the decrease of total NAD level in both tested cell lines (Fig. 5). This fall may also be the reason for depletion of PDHC activity in Ab cells, because NAD is a cofactor of the PDHC E2 subunit (Fig. 4). In SH-SY5Y-DC cells, the PDHC activity was less inhibited by 100 µM ART than in Ab cells, though the cells had a similar rate of NAD decrease (Figs. 4, 5). The explanation for this phenomenon may be the fact that SH-SY5Y cells have a higher rate of oxygen metabolism, and the activity of PDHC in these cells is four times higher than in Ab cells (Fig. 4b).

Our results indicate that different biological forms of tumors responded in a different way to the action of ART. The compound could be an inducer of apoptotic cell death of melanoma, especially the amelanotic form. Our observation, along with the results of others (Stephens and Peacock 1982; Leopold et al. 1987) in relation to the action of acridines on melanoma, could be a basis for the further examination of acridine compounds against the amelanotic form of melanoma. Although the mechanism of the interrelationships between energy metabolism and cells death is not fully understood, interference of ART with the activities of TCA cycle enzymes could be an attractive way to possible therapeutic means.

## Electronic supplementary material

Below is the link to the electronic supplementary material.

Supplementary material 1 (DOCX 18 KB)

Supplementary material 2 (DOCX 16 KB)
