# Supplementary material for: Novel therapeutic compound acridine–retrotuftsin action on biological forms of melanoma and neuroblastoma
Source: J Cancer Res Clin Oncol. 2018 Oct 26;145(1):165–79. doi: 10.1007/s00432-018-2776-4 (PMC6326014; doi:10.1007/s00432-018-2776-4)
Supplement: Supplementary file 1 — Supplementary material 1 (DOCX 18 KB) [file 432_2018_2776_MOESM1_ESM.docx]

**Supplementary Table 1.** The ART (9-RT-1-nitroacridine ) action in comparison to A (9-chloro-1-nitroacridine) on melanoma (melanotic Ma, amelanotic Ab) cells referring regulated cell death features as caspases activation, phosphatidylserine and calreticuline externalization, ROS production and the content of cells with hypodiploid DNA amount (subG0). Values are means ± SD of at least 3 experiments. Statistical analysis by U Mann-Whitney test; Statistically significant change ( p<0.05) in comparison to control values (*) and after incubation with A (**). RT retrotuftsin.

| Percentage of cells with | **Melanotic Ma melanoma** | | | | | | **Amelanotic Ab melanoma** | | | | | |
| --- | --- | --- | --- | --- | --- | --- | --- | --- | --- | --- | --- | --- |
|  | **48 hrs** | | | **72 hrs** | | | **48 hrs** | | | **72 hrs** | | |
|  | **control** | **+A** | **+ART** | **control** | **+** **A** | **+ART** | **control** | **+** **A** | **+ART** | **control** | **+** **A** | **+ART** |
| 1. A**ctivated caspases**  All C+  Early apoptotic C+PI-  Late apoptotic C+PI+ | 7.2.±6.6  1.8±2.6  5.9±5.1 | 4.2±2.8  1.5±2.0  2.7±1.3 | 9.8±2.4  2.8±0.7  7.1±1.7 | 5.5 ±4.2  4.0±4.8  4.0±2.8 | 4.4±3.9  1.7±2.1  2.7±1.9 | 11.4±5.2  3.3±2.5  8.1±3.0 | 9±4.2  2.2±1.9  6.8±2.8 | **15.3±5.0***  **4.6±0.8***  10.7±4.8 | **31.5±7.1*,****  **11.2±5.8***  **20.4±3.2*,**** | 16.3±6.2  4.1±3.4  12.2 ±4.1 | 20.5±7.9  4.8±2.9  15.7±5.5 | **30.3±9.9***  **15.9±10.2***  14.4±4.7 |
| **2**. **Phosphatidylserine externalization**  Early apoptotic An+/PI-  Late apoptotic An+/PI+  Final apoptotic/Necrotic  An-/PI+ | 16.8±8.1  7.9±3.7  4.0 ±4.3 | 14.3±6.1  5.3±1.3  1.9 ±1.4 | 20.9±9.4  11.8±3.1  4.6 ±4.2 | 14.8±6.2  7.0±3.9  2.3± 1.7 | 11.7±7.0  4.6±3.0  16.3±9.6* | **20.6±6.3**  13.3±3.6  3.8± 0.8 | 10.6±5.7  13.9±6.6  5.3±2.6 | 14.0±5.9  19.4±5.7  3.7±1.7 | **24.4±5.6*.****  **29.8±6.8*.****  3.2 ±1.5 | 11.8±7.2  21.8±7.5  3.7 ±2.3 | 15.4±4.0  20.2±4.5  5.9 ±5.2 | **35.3±13.9*.****  22.9±5.6  4.1 ±2.9 |
| **3. Calreticuline (CRT) externalization** | 1.3±0.7 | 0.5±0.2 | 2.0±0.2 | 2.7±3.5 | 0.3±0.2 | 6.9±3.2 | 5.2±4.9 | 2.4±2.5 | 1.1±0.4 | 4.4±2.5 | 2.9±2.2 | 2.4±1.8 |
| **5. Reactive oxygen subtrates (ROS)** | 42.9±8.2 | 41.0±11.4 | 39.9±5.0 | 35.9±12.1 | 38.0±13.8 | 42.7±6.9 | 47.9±23.6 | 61.1±9.7 | 30.3±9.3 | 42.8±20.4 | 53.7±15.5 | 22.2±9.1 |
| **6. sub G0** | 16±7.7 | 17.5±5.5 | 28.6±25.6 | 23.8±11.7 | 23.8±8.4 | 33.1±21.3 | 16.5±7.1 | 17.5±5.2 | **41.3±8.8*,^**^** | 25.6 ±9.5 | 26.5±9.1 | **54.4±20.4*** |
